# Taking hypoglycemic drugs may affect the association between ferritin and nonalcoholic steatohepatitis

**DOI:** 10.1590/1806-9282.20241181

**Published:** 2024-12-02

**Authors:** Rong Hu, Ling-Hai Zeng

**Affiliations:** 1 China Three Gorges University, The People's Hospital, Department of Geriatrics – Yichang, China.

Dear Editor,

We are pleased with the study by Wang et al.^1^, which demonstrates the potential of serum ferritin levels as a serum biomarker for identifying patients with nonalcoholic steatohepatitis and other diseases. Patients, especially men with non-alcoholic fatty liver disease (NAFLD), are at risk for progressive fibrosis. However, I think there are still some issues that need to be addressed.

The main problem with this study is that it does not rule out the possible influence of antidiabetic drugs on the association between ferritin and nonalcoholic steatohepatitis.

Some studies have found that mean serum ferritin levels in uncontrolled T2DM patients were significantly higher than those in controlled T2DM patients^2^. It can be seen that drug intervention can significantly affect serum ferritin in T2DM^3^. Because the included study participants were all patients with T2DM, patients taking antidiabetic medications should be excluded when including study participants.

Another major problem is that the study was unable to eliminate many confounding factors. This study found that participants in the NAFLD group had significantly higher BMI, waist circumference, fasting blood glucose, HbA1c, serum cholesterol, triglycerides, and liver enzymes (AST and ALT) than those in the control group without NAFLD. A large number of studies have found that ferritin is associated with obesity^4,5^, blood lipids^6^, and T2DM^7^.

To sum up, there are many factors that affect serum ferritin levels, such as drugs, obesity, hypertension, and blood lipid levels. When examining the association between ferritin and NAFLD severity, the above-mentioned confounding factors should be excluded.
